# Tai Chi for Overweight/Obese Adolescents and Young Women with Polycystic Ovary Syndrome: A Randomized Controlled Pilot Trial

**DOI:** 10.1155/2022/4291477

**Published:** 2022-06-17

**Authors:** Yan Li, Changle Peng, Meiwei Zhang, Liangzhen Xie, Jinjin Gao, Yingji Wang, Yuanhe Gao, Lihui Hou

**Affiliations:** ^1^First Affiliated Hospital, Heilongjiang University of Chinese Medicine, Harbin, China; ^2^Graduate School, Heilongjiang University of Chinese Medicine, Harbin, China; ^3^College of Pharmacy, Harbin Medical University, China

## Abstract

**Background:**

Exercise is one of the recommended interventions for polycystic ovary syndrome (PCOS), and current evidence has shown that Tai chi may have favorable effects. The objective of this randomized controlled pilot trial was to study the feasibility and potential effects of Tai chi for overweight/obese adolescents and young women with PCOS, so a future definitive randomized controlled trial (RCT) can be well designed and implemented.

**Materials and Methods:**

This study recruited 50 patients who were randomly assigned to two groups (Tai chi and self-monitored exercise) at a ratio of 3 : 2. The intervention lasted for 3 months, and the feasibility and effectiveness outcomes were measured.

**Results:**

A total of 42 patients completed the study, including 24 in the Tai chi group and 18 in the control group. Compared with the self-monitored exercise group, there was a significantly decreased body mass index (BMI) in the Tai chi group adjusted for baseline BMI. The testosterone level and lipid profile were also decreased compared to controls; the same tendency was also observed for the homeostasis model assessment of insulin resistance (HOMA-IR), but the difference did not achieve statistical significance. Twenty-four (out of 30, 80%) patients in the Tai chi group and 18 (out of 20, 90%) patients in the self-monitored exercise group completed the data collection. A total of 36 exercise sessions were held in both groups. Patients in the Tai chi group took a mean of 34.0 ± 2.21 classes (93.06%), and those in the self-monitored exercise group engaged in 32 ± 3.06 exercise sessions (88.27%) out of the 36 required exercise sessions.

**Conclusions:**

The present pilot study was feasible to deliver; there was a decrease in BMI, testosterone level, and lipid profile for PCOS patients in the Tai chi group at 3 months. In a future definitive trial, lower recruitment rate and outcome measurements lead to poor patient acceptance such as the 5-time point oral glucose tolerance test need to be considered and one fixed type of aerobic exercise and supervision from the investigator for the control group are also needed. Trial registration: ClinicalTrials.gov, NCT02608554.

## 1. Background

Polycystic ovary syndrome (PCOS) is one of the most prevalent endocrine disorders, and it affects 5–20% of reproductive-age women [1]. The clinical characteristics of PCOS include both reproductive features and metabolic features [2, 3], and being overweight/obese worsens all clinical features of PCOS.

Treatment aims in PCOS include achieving a healthy weight, improving any underlying hormonal disturbances, preventing future reproductive and metabolic complications, and improving the quality of life [4]. Lifestyle interventions, including dietary, exercise, behavioral, or their combination, are recommended as first-line management for PCOS patients [4]. A 5% to 10% weight loss in PCOS patients is considered clinically significant and is associated with metabolic, reproductive, and psychological health benefits [4]. A Cochrane systematic review reported that lifestyle treatment might result in a modest reduction in weight and an improvement in abdominal obesity, namely, a mean difference in weight for lifestyle changes compared to minimal treatment of around 1.68 kg [5]. Several published systematic reviews suggested that there is limited evidence concerning exercise alone on reproductive outcomes; however, exercise may have favorable effects on body composition and insulin resistance [6–10].

Tai chi is an exercise system that follows Chinese medicine theory and has been practiced in China since the seventeenth century. The five styles which are practiced most commonly today are the Yang, Chen, Wu, Sun, and Woo styles [11]. In the past few decades, an increasing number of studies have focused on its beneficial effects in treating diseases and maintaining health, and it has received increasing attention in the West [12, 13]. The physiological and psychosocial benefits of Tai chi on chronic diseases that are very closely related to PCOS, such as obesity, cardiovascular diseases (CVDs), and type 2 diabetes [14–16], have been confirmed.

Although exercise is recommended as one of the first-line approaches for the treatment of PCOS, specific recommendations for exercise prescription are lacking, and further research into these fields is necessary. We performed this study to address whether a randomized controlled trial (RCT) of Tai chi in treating overweight/obese adolescents and young women with PCOS was an appropriate trial design and was feasible with regard to (i) recruitment and retention and (ii) treatment fidelity. In addition, we wished to assess the potential effectiveness of a 3-month Tai chi intervention and collect and synthesis data, from which the sample size of a definitive RCT could be estimated. This pilot trial may not be adequately powered for assessing effectiveness; however, together with the feasibility outcomes, the results obtained will provide data to estimate the parameters required to design a definitive RCT in the future.

## 2. Methods

### 2.1. Trial Design

The present study was a single-blind (assessors), parallel randomized clinical pilot trial. Unequal randomization of 3 : 2 was adopted since it has been suggested that it is better to have as many participants receive the intervention as is feasible [17]. The present study was registered at ClinicalTrials.gov (NCT02608554) and compliant with Consolidated Standards of Reporting Trials (CONSORT) [18] and CONSORT extension to randomized pilot and feasibility trials [17]. The study was conducted in accordance with the Declaration of Helsinki and received approval from the First Affiliated Hospital of the Heilongjiang University of Chinese Medicine Institutional Review Boards (approval number: HZYLLKT201500201). The details of the trial protocol were reported in another paper [19].

### 2.2. Participants

The present study was conducted in the First Affiliated Hospital, Heilongjiang University of Chinese Medicine. Patients were recruited from the Gynecology Outpatient Clinic. All study visits and Tai chi interventions took place at the abovementioned hospital. Self-monitored exercises were held in a home-based environment.

Inclusion criteria were used as follows: (i) women aged between 18 and 35 years [20]; (ii) patients who confirmed diagnosis of PCOS according to the modified Rotterdam criteria, and all subjects had anovulation plus either polycystic ovaries and/or hyperandrogenism; (iii) patients with at least two years after menarche; (iv) patients whose body mass index (BMI) is equal to or greater than 23 kg/m^2^ [21]; and (v) patients with no desire to have children within 6 months. PCOS was defined by the modified Rotterdam criteria as oligomenorrhea or amenorrhea, together with the presence of ≥12 antral follicles (≤9 mm) and/or ovarian volume >10 mL on transvaginal scanning, and/or clinical/biochemical hyperandrogenism. Oligomenorrhea was defined as an intermenstrual interval >35 days and less than eight menstrual bleeds in the past year. Amenorrhea was defined as an intermenstrual interval >90 days. Clinical hyperandrogenism in mainland China is defined as Ferriman–Gallwey (FG) score ≥5 [22].

Exclusion criteria were as follows: (i) administration of other medications known to affect the reproductive function or metabolism within the past 3 months, including oral contraceptives, gonadotropin-releasing hormone (GnRH) agonists and antagonists, antiandrogens, gonadotropins, antiobesity drugs, Chinese herbal medicines, and antidiabetic drugs, such as metformin and thiazolidinediones, somatostatin, diazoxide, and calcium-channel blockers; (ii) patients with other endocrine disorders, including 21-hydroxylase deficiency, hyperprolactinemia, uncorrected thyroid (including hypothyroidism, hyperthyroidism, and/or thyroid autoimmunity) disease, and suspected Cushing's syndrome; and (iii) patients with known severe organ dysfunction or mental illness.

### 2.3. Interventions

The 24 forms of the simplified Tai chi program (a modified Yang style) recommended as a health-benefiting sport by the General Administration of Sport of China were applied [23]. Patients attended a 60 min exercise session three times per week for 12 weeks, with an intensity based on their original level of physical activity. Each session comprised 40 min of Tai chi training plus a 10 min warm-up and cool-down. Tai chi training was instructed by experienced Tai chi instructors who were qualified in teaching. Patients in the self-monitored exercise group were asked to add extra exercise in addition to their routine exercise. Self-monitored exercise consisted of brisk walking, cycling, jogging, or any other aerobic exercise for 60 min, three times per week for 12 weeks [24]. Adherence to the exercise was tracked via self-reported logs.

### 2.4. Outcome Measurements

The primary outcome was the BMI change from baseline. BMI was calculated using weight (kg)/height (m^2^). The secondary outcomes included (i) homeostasis model assessment of insulin resistance (HOMA-IR), fasting plasma glucose (FPG), and fasting insulin (FINS); (ii) hormone profile including testosterone (T), androstadiendione (AND), sex hormone-binding globulin (SHBG), dehydroepiandrosterone sulfate (DHEAS), follicle-stimulating hormone (FSH), luteinizing hormone(LH), and oestradiol (E_2_); (iii) fasting-lipid metabolic profile: triglycerides (TG), cholesterol (TC), high-density lipoprotein cholesterol (HDL-C), and low-density lipoprotein cholesterol (LDL-C); (iv) the weight, waist-to-hip ratio, and FG score before and after treatment; (v) feasibility outcomes including participant recruitment rates, retention rates, and treatment fidelity monitored through attendance records; and (vi) adverse events. We failed to collect data from all participants including oral glucose tolerance tests (OGTTs), blood pressure, or the presence of acne because a large proportion of the enrolled patients refused to undergo these examinations.

### 2.5. Randomization and Allocation Concealment

After the baseline evaluation, 50 subjects will be allocated randomly into one of the two groups in a ratio of 3 : 2. The identification code and random number, which are unique for each subject, were generated using SAS 9.2 by an independent agency (TCM Translational Medicine Research Center, First Affiliated Hospital, Heilongjiang University of Chinese Medicine). These assignments were put into sealed and opaque envelopes. The envelopes will only be opened after the subject has completed baseline clinical assessments.

### 2.6. Blinding

The present trial is a single-blinded trial. Outcome assessors and people responsible for statistical analysis will be blinded to the randomization status.

### 2.7. Statistical Analysis

The effect size of Tai chi intervention on PCOS was not available by the time when this study was designed. As this trial is an exploratory study mainly to evaluate the feasibility of a large clinical trial, sample size was determined by recommendations for a pilot trial, and *n* = 30 was recognized as a reasonable minimal sample size for pilot studies [25,26]. For such a trial designed with 90% power and two-sided 5% significance, the sample size was set to 50 patients with an allocation ratio of 3 : 2 as described in the protocol [19], and the standardized effect sizes is medium (0.5) [27].

All analysis was performed using SAS version 9.3. Data were summarized using means (±SDs) for continuous variables. An analysis of covariance (ANCOVA) model was used to compare the mean changes in outcomes from baseline to the end of the intervention, including baseline measurement covariates. Comparisons between the groups were made using the *t*-test (two-tailed) or the Mann–Whitney *U* test. A paired *t*-test or the Wilcoxon rank test was used to compare the variables from the baseline to the end of the intervention. *P* < 0.05 was considered statistically significant.

## 3. Results

**Figure 1:**
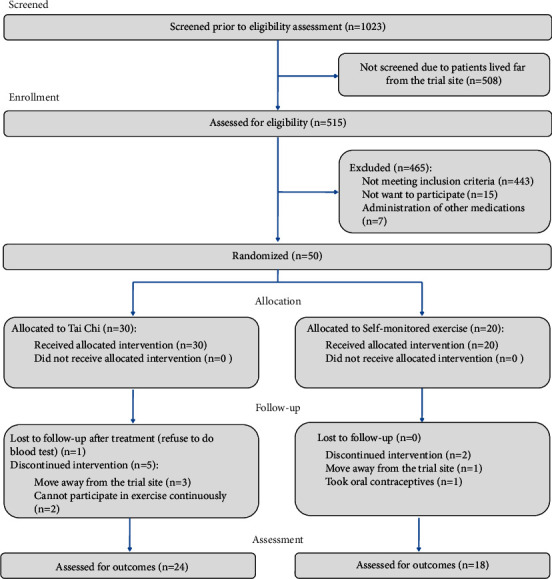
Flow diagram of the participants through the study.

Fifty patients were enrolled in the trial; 30 were randomized to the Tai chi group and 20 to the control group. Eight patients terminated the study prematurely; thus, 42 patients completed the end of treatment assessments and were included in analysis (Figure 1). Their anthropometric data and baseline characteristics are shown in Table 1.

### 3.1. Primary Outcome

The primary outcome measurement was the BMI change from baseline. After 3 months of treatment, BMI significantly decreased in the Tai chi group compared with before treatment. However, BMI did not differ before and after treatment in the self-monitored exercise group. Compared with the self-monitored exercise group, there was a significantly decreased BMI in the Tai chi group adjusted for baseline BMI (Table 2).

### 3.2. Secondary Outcome

After treatment, the Tai chi group showed favorable changes in body weight, FINS, and HOMA-IR with no significant differences between the groups. For the hormonal profile, *T* significantly decreased in the Tai chi group compared with the self-monitored exercise group. In addition, Tai chi showed a significant effect in alleviating lipid profile disorders, including decreased TG, TC, and LDL-C, compared with the self-monitored exercise group (Table 3).

Recruitment was conducted in the Gynecology Outpatient Clinic, First Affiliated Hospital, Heilongjiang University of Chinese Medicine. A total of 1023 subjects were screened prior to eligibility assessment, and we excluded 508 of them since these subjects lived far away from the trial site and could not participate in Tai chi exercise under supervision. A total of 515 subjects were assessed for eligibility, 443 were excluded for not meeting inclusion criteria, 15 were excluded because they did not want to participate in the trial, and 7 were excluded for taking other medications that could have affected the outcome. The primary barriers for participating in this study were patients who lived too far away from the hospital or had no time to participate due to work or household activities.

Fifty patients were enrolled in the trial. After three months of intervention, 24 (of 30, 80%) patients in the Tai chi group and 18 (of 20, 90%) patients in the self-monitored exercise group completed the data collection. Reasons for dropping out are recorded in Figure 1.

A total of 36 Tai chi classes were held during the intervention, and patients attended a mean of 34.0 ± 2.21 classes (93.06%). Patients in the self-monitored exercise group undertook 32 ± 3.06 exercise sessions (88.27%) out of 36 required exercise sessions.

### 3.3. Adverse Events

There were no adverse events reported during the study period.

## 4. Discussion

In this study, we designed a pilot RCT to assess the feasibility and potential effectiveness of Tai chi in treating overweight/obese adolescents and young women with PCOS. The main results showed that the major impediment to recruitment was a long distance from the trial site and no time to exercise, which were also the main reasons for dropping out. However, the trial also found that Tai chi may significantly decrease the BMI, T level, and lipid profile; the same tendency was also observed for HOMA-IR, but the difference did not achieve statistical significance compared to self-monitored exercise. Since this pilot trial was intentionally designed without adequate power to test the efficacy, these effectiveness outcomes should be interpreted with caution.

The recruitment rate of this pilot study seemed lower than that in other exercise studies in PCOS [28, 29]. The barriers to recruitment could partially be because, in this pilot trial, Tai chi needed to be practiced under the guidance of an experienced instructor and only subjects who lived near the clinical site and who were available during the exercise sessions could participate. However, this situation may have little effect when Tai chi is used as a treatment because after mastering Tai chi practice, patients can exercise on their own, which improves the flexibility of time and location. And, online learning will also be a strategy to promote more patients to involve. The retention in the Tai chi group was similar to that in the self-monitored exercise group and other reports [28, 30], which suggests that Tai chi may be a feasible intervention for women with PCOS. Five-time point OGTT had low acceptance to patients, especially at the end of treatment visit. Acne was difficult to document precisely because patients were not willing to remove makeup. Blood pressure was not fully documented because these young patients thought it was irrelevant to the condition.

BMI will be used as the primary outcome measure for the future definitive trial. Data from the present trial showed that the BMI value in the Tai chi and control group was 27.39 ± 3.58 m/kg^2^ and 29.62 ± 3.95 m/kg^2^, respectively. Sixty-six participants per group will be required to achieve a 90% statistical power (*α* = 0.05) for changes between the intervention group and the control group. With an estimated 20% attrition, we plan to recruit a total of 160 participants.

Tai chi has been reported to alleviate the lipid profile disorders in other conditions [31–34], which was also observed in this trial. A significant difference was observed between the two intervention groups in TC, TG, and LDL-C. BMI and HOMA-IR were also decreased in the Tai chi group, which seems consistent with reports that Tai chi could contribute to weight loss and improve insulin resistance [35], and the effect seemed similar to other aerobic exercises [36]. Aerobic exercises could improve insulin measures in women with PCOS, especially those involved in more vigorous activity and/or more frequent weekly exercise or sessions of longer duration [37]. Vigorous aerobic activity at least three days per week for 30 min or more is recommended. A heart rate monitor or maximal oxygen consumption (VO_2max_) guided intensity levels (∼60% VO_2max_) are advised to obtain insulin-related benefits [37].

Little is known about the effect of Tai chi on sex hormones. In this trial, we observed a decrease in *T* levels in the Tai chi group. Current evidence has shown that improvements in androgens are more likely with resistance or strength training instead of aerobic exercise [29, 37]. Yoga, another popular mind-body exercise, has also been reported to improve *T* levels [38]. Additional studies are warranted to confirm these findings.

As a treatment for PCOS, it is suggested that a minimum of 120 minutes of vigorous intensity exercise per week is necessary to provide favorable health outcomes [6]. A systematic review compared the effects of high-intensity interval training (HIIT) and moderate-intensity steady-state exercise (MISS) in PCOS patients [37]. The authors reported that neither exercise type improved HOMA-IR, MISS improved the relative maximal oxygen consumption and anthropometric profile, and decreased the TC (moderate-quality evidence), and MISS exercise appeared to be more effective in improving cardiorespiratory fitness and BMI in women with PCOS than HIIT [39].

The exercise prescription of Tai chi still needs future investigation, specifically in terms of exercise intensity, frequency, and duration. It has been shown that the intensity of Tai chi is approximately 52–63% of the heart rate range [40], which is comparable to low- or moderate-intensity aerobic exercises [41–43]. The intensity of Tai chi varies depending on the training style and posture [11]. The Yang style is the most popular due to its gentleness and extensive stretching; the Chen style requires more strength and involves more skipping movements, which may consume the most energy among all five branches [44]. A recently published systematic review compared different training styles of Tai chi among patients with type 2 diabetes, and the results showed that different styles could result in variable effectiveness [45]. The most suitable form of Tai chi for PCOS still needs further study. We also noticed that there are studies of modified Tai chi [46–48] or Tai chi combined with other exercises (e.g., theraband) [49, 50]. These modifications may point to a potential strategy for treating PCOS patients.

It has been confirmed that exercise programs that incorporate ∼170 min of exercise/weekly improved insulin sensitivity more than a program utilizing ∼115 min of exercise/weekly among PCOS patients [51]; therefore, practicing Tai chi at least 3 times per week may be suitable, which was well accepted in the present pilot trial. However, current evidence is insufficient to support whether long-term Tai Chi training is superior to short-term training [45]. Another point also needs to be elucidated, that is, what is the optimal time of day to exercise? A crossover study of HIIT for type 2 diabetes showed that afternoon HIIT was associated with better glucose control than morning HIIT [52]. Additional studies are necessary to elucidate the exercise prescription of Tai chi.

Given the preliminary nature of a feasibility study, the present study has several limitations. First, this trial only enrolled overweight/obese women with PCOS, and the effects of Tai chi need future investigation in normal-weight subjects, and reproductive outcomes, such as the live birth rate and the ovulation rate, need to be considered. Second, the present trial did not investigate the impact of Tai chi on quality of life. As a mind-body exercise, Tai chi is reported to have favorable effects in improving the quality of life of the subjects [53–55], and it is well known that PCOS is associated with a lower quality of life compared to healthy women [56–59]. Studies in this area should be carried out in the future. Last but not the least, we used multiple aerobic exercises in the control group without supervision from the investigator, this may cause bias between the groups. A good strategy of supervision from investigators or quantitative measurements of exercise intensity are needed in the future.

## 5. Conclusions

Tai chi is a mind-body approach that may be considered a therapeutic option in the multidisciplinary management of PCOS, and the present pilot study was feasible to deliver. There was a decrease in BMI, testosterone level, and lipid profile for PCOS patients in the Tai chi group at 3 months. In a future definitive trial, lower recruitment rate may be fixed by online teaching, a 5-time point oral glucose tolerance test which leads to poor patient acceptance need to be considered, one fixed type of aerobic exercise and supervision from the investigator for the control group are needed, and quantitative measurement of exercise intensity will also be considered. The evidence generated from the present pilot trial will inform further definitive trials that are required to evaluate the effectiveness of Tai chi on PCOS and to develop optimized exercise prescriptions.

## Figures and Tables

**Table 1:** Characteristics of the study participants.

	Tai chi group (*n* = 24)	Self-monitored exercise group (*n* = 18)
Age (years)	23.2 ± 4.38	22.9 ± 4.64
Weight (kg)	74.99 ± 11.62	78.33 ± 13.45
Height (m)	1.62 ± 0.05	1.63 ± 0.05
BMI (m/kg^2^)	28.41 ± 4.03	29.57 ± 4.47
Waist circumference (cm)	97 ± 8.73	96.4 ± 13.67
Hip circumference (cm)	107.27 ± 7.9	106.95 ± 8.05
Waist-to-hip ratio (cm/cm)	0.9 ± 0.05	0.9 ± 0.08

Data are expressed as the mean ± standard deviation. BMI: body mass index; FG score: Ferriman–Gallwey score.

**Table 2:** Primary outcome measure.

	Tai chi group (*n* = 24)		*P* value	Self-monitored exercise group (*n* = 18)		*P* value	Differences in changes between the groups at 3 months (*P* value)
	Baseline	3 months		Baseline	3 months		
BMI (m/kg^2^)	28.41 ± 4.03	27.39 ± 3.58	0.008^Δ^	29.57 ± 4.47	29.62 ± 3.95	0.524	0.003^*∗*^

Data are expressed as the mean ± standard deviation. BMI: body mass index; ^*∗*^indicates *P* < 0.05 vs. the self-monitored exercise group; ^Δ^ indicates *P* < 0.05 vs. baseline.

**Table 3:** Characteristics of study participants.

	Tai chi group (*n* = 24)		*P* value	Self-monitored exercise group (*n* = 18)		*P* value	Differences between the groups at 3 months (*P* value)
	Baseline	3 months		Baseline	3 months		
Weight (kg)	74.99 ± 11.62	73.08 ± 11.55	0.009^Δ^	78.33 ± 13.45	77.97 ± 12.1	0.547	0.203
Waist-to-hip ratio (cm/cm)	0.9 ± 0.05	0.9 ± 0.04	0.956	0.9 ± 0.08	0.91 ± 0.08	0.481	0.833
FPG (mmol/L)	4.89 ± 0.89	4.84 ± 0.54	0.542	4.79 ± 0.53	4.83 ± 0.38	0.997	0.964
FINS (*μ*U/ml)	20.08 ± 12.39	13.99 ± 8.2	0.035^Δ^	19.35 ± 10.26	20.36 ± 14.1	0.275	0.073
HOMA-IR	4.51 ± 3.15	3.05 ± 1.82	0.042^Δ^	4.07 ± 2.0	4.36 ± 3.12	0.396	0.094
T (ng/dL)	61.84 ± 20.96	49.89 ± 15.77	0.045^Δ^	63.92 ± 26.49	66.59 ± 28.15	0.822	0.032^*∗*^
AND (ng/mL)	274.21 ± 146.29	312.71 ± 182.51	0.53	285.3 ± 146.62	296.83 ± 136.46	0.892	0.758
SHBG (nmol/L)	19.34 ± 10.78	22.74 ± 9.64	0.093	28.61 ± 37.42	23.24 ± 13.73	0.512	0.890
DHEAS (*μ*g/dl)	7.17 ± 5.26	5.16 ± 2.45	0.017^Δ^	5.92 ± 2.66	4.98 ± 2.51	0.01^Δ^	0.818
LH (mIU/mL)	9.25 ± 5.08	4.54 ± 1.45	0.09	11.11 ± 12.01	4.42 ± 1.1	0.253	0.779
FSH (mIU/mL)	4.54 ± 1.66	6.96 ± 3.6	0.748	4.45 ± 0.99	7.7 ± 5.01	0.882	0.581
LH/FSH	2.19 ± 1.09	1.58 ± 0.74	0.024^Δ^	2.28 ± 1.65	1.74 ± 1.11	0.232	0.556
E_2_ (pg/mL)	58.81 ± 20.36	47.99 ± 18.42	0.052	62.2 ± 54.92	59.85 ± 19.16	0.68	0.049^*∗*^
TG (mmol/L)	1.79 ± 1.35	1.33 ± 0.61	0.308	2.29 ± 1.29	2.3 ± 1.25	0.475	0.006^*∗*^
TC (mmol/L)	4.13 ± 1.09	3.81 ± 0.66	0.431	4.78 ± 1.18	4.75 ± 1.32	0.585	0.11^*∗*^
HDL-C (mmol/L)	1.07 ± 0.28	1.07 ± 0.27	0.936	1.11 ± 0.21	1.12 ± 0.28	0.86	0.572
LDL-C (mmol/L)	2.63 ± 0.71	2.43 ± 0.65	0.368	3.01 ± 0.99	3.13 ± 0.99	0.893	0.009^*∗*^
FG score (score)	5.0 ± 2.52	4.71 ± 2.93	0.206	4.35 ± 2.58	5.0 ± 3.27	0.327	0.817

^*∗*^indicates *P* < 0.05 vs. the self-monitored exercise group; ^Δ^indicates *P* < 0.05 vs. baseline; data are expressed as the mean ± standard deviation. FPG: fasting plasma glucose; FINS: fasting insulin; HOMA-IR: homeostasis model assessment of insulin resistance; T: testosterone; AND: androstadienone; SHBG: sex hormone-binding globulin; DHEAS: dehydroepiandrosterone sulfate; LH: luteinizing hormone; FSH: follicle-stimulating hormone; E_2_: oestradiol; TC: cholesterol; TG: triglycerides; HDL-C: high-density lipoprotein cholesterol; LDL-C: low-density lipoprotein cholesterol; FG score: Ferriman–Gallwey score.

## Data Availability

The datasets used to support the findings of this study are available from the corresponding author upon request.

## Abbreviations

PCOS: Polycystic ovary syndrome
RCT: Randomized controlled trial
BMI: Body mass index
HOMA-IR: Homeostasis model assessment of insulin resistance
CONSORT: Consolidated Standards of Reporting Trials
FG: Ferriman–Gallwey
GnRH: Gonadotropin-releasing hormone
FPG: Fasting plasma glucose
FINS: Fasting insulin
T: Testosterone
AND: Androstadiendione
SHBG: Sex hormone-binding globulin
DHEAS: Dehydroepiandrosterone sulfate
FSH: Follicle-stimulating hormone
LH: Luteinizing hormone
E_2_: Oestradiol
TG: Triglycerides
TC: Cholesterol
HDL-C: High-density lipoprotein cholesterol
LDL-C: Low-density lipoprotein cholesterol
OGTTs: Oral glucose tolerance tests
ANCOVA: Analysis of covariance
VO_2max_: Maximal oxygen consumption
HIIT: High-intensity interval training
MISS: Moderate-intensity steady-state exercise.

## Supplementary Materials

Supplementary Materials CONSORT 2010 checklist of information to include when reporting a randomized trial.
